# Long‐Term in vivo Observation of Maize Leaf Xylem Embolism, Transpiration and Photosynthesis During Drought and Recovery

**DOI:** 10.1111/pce.15414

**Published:** 2025-02-03

**Authors:** Brendan S. Allen, Jared J. Stewart, Stephanie K. Polutchko, Troy W. Ocheltree, Sean M. Gleason

**Affiliations:** ^1^ Water Management and Systems Research Unit, USDA‐ARS Fort Collins Colorado USA; ^2^ Department of Forest and Rangeland Stewardship Colorado State University Fort Collins Colorado USA; ^3^ Department of Ecology & Evolutionary Biology University of Colorado Boulder Colorado USA

**Keywords:** drought, embolism, leaf shrinkage, maize, photosynthesis, xylem

## Abstract

Plant water transport is essential to maintain turgor, photosynthesis and growth. Water is transported in a metastable state under large negative pressures, which can result in embolism, that is, the loss of function by the replacement of liquid xylem sap with gas, as a consequence of water stress. To avoid experimental artefacts, we used an optical vulnerability system to quantify embolism occurrence across six fully expanded maize leaves to characterize the sequence of physiological responses (photosynthesis, chlorophyll fluorescence, whole‐plant transpiration and leaf inter‐vein distance) in relation to declining water availability and leaf embolism during severe water stress. Additionally, we characterize the recovery of leaf function in the presence of sustained embolism during a 6‐day recovery period. Embolism formation occurred after other physiological processes were substantially depressed and were irreversible upon rewatering. Recovery of transpiration, net CO_2_ assimilation and photosystem II efficiency were aligned with the severity of embolism, whereas these traits returned to near pre‐stress levels in the absence of embolism. A better understanding of the relationships between embolism occurrence and downstream physiological processes during stress and recovery is critical for the improvement of crop productivity and resilience.

## Summary Statement

Xylem embolism, transpiration and photosynthesis were observed non‐invasively and continuously in maize leaves during prolonged drought stress and subsequent recovery. The extent of embolism was aligned with irreversible declines in transpiration and photosynthesis after re‐watering.

## 1 Introduction

Water movement within plants is essential for critical functions, including CO_2_ assimilation, growth and turgor. Water moves from the roots to the sites of photosynthesis through xylem conduits under negative pressure. The high tension inherent in these conduits can result in embolism formation, which are gas blockages disrupting the transport of liquid water (Sperry and Tyree 1988; Tyree et al. 1986). Embolism formation introduces hydraulic resistances within the xylem system, which impacts leaf‐level water utilization and recovery after re‐watering (Gleason et al. 2017b; Nadal et al. 2023). The interconnectivity of traits influencing water transport and utilization forms a finely tuned network shaped by both natural and artificial selection (Brodribb and Holbrook 2003; Carlquist 2012; Cochard 2002; Gleason 2015). Understanding the relationship between embolism and leaf physiology is critical for ensuring resilient and productive agricultural practices in the face of evolving environmental challenges.

Understanding whole‐plant dynamics during the terminal stages of drought stress is critical to making meaningful advances in drought resilience for agricultural crop species. Most crop physiological research has focused on improvement of stomatal and photosynthetic functioning and/or root traits (timing, magnitude, response to soil and climate cues; Ainsworth and Ort 2010; Lopes et al. 2011; Palta and Turner 2019; White 2019) without consideration for the integrity of the liquid water transport pathway, which is vulnerable during drought (Brodribb, Feild, and Jordan 2007; Gleason 2015). It is, therefore, critical that we know the *relative* importance of these thresholds, the connections between them, as well as their recovery (or lack of) after drought so crop improvement programmes can properly prioritize the most likely trait networks that confer improved performance (Gleason et al. 2022). Leaves, being the primary sites of evaporation, and susceptible to variation in light and evaporative demand, are often the first organs to experience detrimental effects due to embolism (Song et al. 2022). By investigating the implications of embolism accumulation, we aim to shed light on the intricate canopy dynamics of leaf response and recovery to water stress.

Failure of the hydraulic transport systems caused by xylem embolism is thought to be a critical point to plant growth and survival, having profound consequences if not reversed (i.e., conduit refilling). Previous studies have made claims that maize, among other species, can experience embolism formation during the day and subsequently refill embolism overnight. Many of these studies, in addition to measurements of hydraulic conductivity, have relied on the excision of stems or roots (Gleason et al. 2017a; McCully 1999), or the monitoring of acoustic emissions (Tyree et al. 1986), which have resulted in the conclusion that embolism formation and repair may occur within the range of common midday conditions (i.e., midday leaf water potentials ranging ca. −1.5 to −2.0 MPa). However, few studies have investigated the timing, spatial patterns and the water potentials associated with leaf xylem embolism in intact plants (Cochard 2002; Hwang, Ryu, and Lee 2016; Ryu, Hwang, and Lee 2016). It is critical that we focus on the utilization of intact plants to make meaningful conclusions of when and under what conditions embolism formation occurs. This is necessary because methods that require the cutting/removal of plant organs have likely skewed our understanding of the xylem pressures and tissue water contents associated with xylem embolism and decline in hydraulic conductivity (Cochard and Delzon 2013; Silva et al. 2024; Torres‐Ruiz et al. 2015; Wheeler et al. 2013) leaving uncertainty about the series of unfortunate events that lead to leaf or plant mortality. This prompted our use of non‐destructive methodology focused on identifying water potentials associated with key physiological thresholds in this important species, such as stomatal closure, decline in photosystem II (PSII) efficiency, embolism and embolism reversal (if observed). Additionally, we sought to understand the relative timing of these thresholds during prolonged drought, as well as the timing of their recovery after plants were returned to a fully hydrated state. By investigating the implications of embolism accumulation in fully intact plants, we aim to shed light on the intricate dynamics of the leaf response to water stress.

Leaf shrinkage has garnered attention for its potential implications in drought stress responses, being relatively easy to measure as it can be an added utility when using optical methods (Canny et al. 2012; Johnson, Jordan, and Brodribb 2018; Scoffoni et al. 2014). Water released from shrinking tissues can be quantitatively linked to the delay of embolism and leaf death after stomatal closure (Blackman et al. 2016; Gleason et al. 2014). Connecting leaf shrinkage, an easily measurable trait, with physiological processes could allow for easier identification of critical thresholds during drought periods, especially when other measures of plant function (e.g., net CO_2_ assimilation and transpiration) have already reached depressed steady states lacking informative resolution (Bourbia, Lucani, and Brodribb 2022).

This study aims to identify the occurrence of leaf embolism, particularly in relation to the decline of essential leaf functions and physiological processes when plants are subjected to sustained low water potentials as a result of diminishing water availability. We conducted this investigation in laboratory conditions focused on identifying leaf responses to desiccation across canopy leaves during a controlled dry down and well‐watered recovery of potted maize plants. We hypothesized that the recovery of leaf‐level physiological traits (net CO_2_ assimilation, stomatal conductance, PSII efficiency and inter‐vein distance) would be negatively correlated with the degree of embolism sustained during desiccation. We also predicted that essential physiological functions would decline with water availability. Furthermore, we predicted that the relative timing of decline in physiological traits and the occurrence of embolism would be similar among leaf positions in the canopy.

## 2 Materials and Methods

### 2.1 Plant Material and Growth Conditions

*Zea mays* L. subsp. *mays* genotype B73 was grown from seed (obtained from USDA‐ARS Germplasm Resources Information Network; accession PI 550473) under greenhouse conditions (see below) at the USDA Crops Research Laboratory, Fort Collins, CO, USA. The use of B73 was deliberate due to its fully sequenced genome, aligning with our broader objective of identifying traits that support crop resilience by establishing meaningful benchmarks for plant stress in this model genotype. A drip‐line irrigation system, regulated by a battery‐operated timer (RBC7000, DIG, Corp., Vista, CA, USA) connected to 1.9‐cm diameter polytubing, delivered water to the plants at regular 12‐h intervals. Plants were grown in 15 L nursery pots filled with Profile Greens Grade (PROFILE Products LLC, Buffalo Grove, IL), a nonnutritive calcined clay substrate with favourable water retention properties. Nutrients were provided by 15 g of Osmocote Plus slow‐release fertilizer (The Scotts Miracle‐Gro Company, Marysville, OH, USA), which was mixed homogeneously into the substrate.

**Figure 1:**
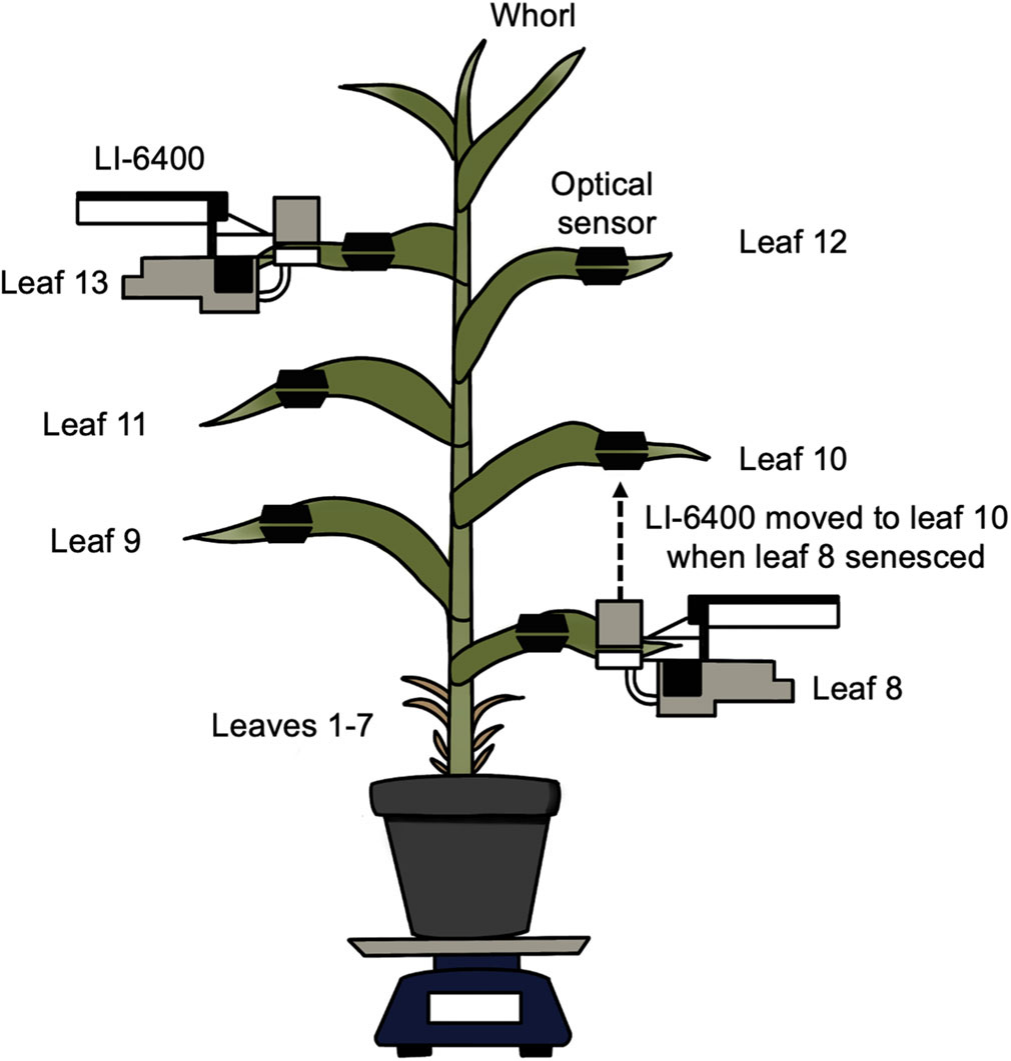
Schematic diagram of experimental setup.

After approximately 7 weeks of growth (i.e., 50 days after seeds were sown), plants were moved to laboratory conditions and placed in a custom‐built growth cage (Figure 1 and Supporting Information S1: Figure S1; see also Gleason et al. 2024) with a 15‐h photoperiod (05:30 to 20:30) provided by two rows of Philips GreenPower LED top lighting (maximal light intensity of 600 µmol photons m^−2^ s^−1^). The relative humidity was 19.2 ± 11.9%, temperature was 24.5 ± 0.6°C and vapour pressure deficit (VPD) was 2.49 ± 0.39 kPa. Once plants were in the lab space, water was withheld until xylem embolism was observed in the majority of upper leaves (typically representing some embolism formation in three out of six instrumented leaves). This range in leaf xylem embolism was intentional, as we aimed to have a gradient of embolism severity present across canopy leaves to examine the effect of embolism on the speed and completeness of recovery after plants were re‐watered. At the time of rewatering, approximately 3 L of water was added to each pot to saturate the substrate. Plants remained well watered for the duration of the 6 day recovery period by the daily replacement of equivalent amounts of water lost through transpiration.

### 2.2 Water Potential

Plants were grown in four cohorts of eight plants, with one plant from each cohort being selected for instrumentation and non‐destructive measurements (Figure 1; see below). The other plants were subjected to periodic water potential measurements using a Scholander‐type pressure chamber (1505‐D, PMS Instruments, Albany, OR, USA). For some plants, water potential was measured from every expanded leaf available at a single time point. This spatial variability made it difficult to assign a single water potential to the whole plant because leaves were found to become hydraulically isolated from the rest of the plant as they desiccated. Therefore, leaf water potential was estimated for each leaf based on pressure chamber measurements taken from companion plants and predicted using a linear regression, with time as the independent variable (Supporting Information S1: Figure S2).

### 2.3 Leaf Optical Measurements

Embolism occurrence and changes in inter‐vein distance were assessed using an optical vulnerability system based on methods described by Brodribb et al. (2016), as modified by Gleason et al. (2024). The use of an optical vulnerability system allowed us to pair leaf embolism with measurements made from adjacent gas exchange cuvettes during the dry down and recovery periods. Six optical sensors were positioned approximately 15–30 cm from the tip of the six most recently collared leaves (i.e., Leaves 8–13 from the bottom of the plant; Figure 1). Each optical sensor imaged a leaf area of approximately 16 cm^2^. The adaxial side of the leaf was loosely held flat against a microscope slide at the imaging plane. A 3D‐printed clear resin diffuser plate, with a channel for the midrib, was positioned between the LED light and the leaf, stabilizing the leaf during experimentation but still allowing for leaf shrinkage. Optical sensors were secured with aluminium T‐slot framing clamped to the camera housing. Generic 5 W white LEDs were placed in the centre of the light housing to provide light only during the imaging process. USB cameras, 2MP OV2710 (Arducam Technology Co., Kowloon, Hong Kong, PRC) were used to image the adaxial face of the leaf. Images were taken in 5‐min intervals using OpenCV‐Python controlled by a computer running Ubuntu Linux (22.04 LTS).

The image stacks generated by the optical sensors were evaluated for leaf vein embolization using ImageJ software (Schneider, Rasband, and Eliceiri 2012). Inter‐vein distance was calculated by taking a normalized difference in pixel positions of the leaf major veins on either side of the midrib and expressing values as a percent change from the initial distance between leaf veins (Patil et al. 2022). Embolism was expressed as a ratio of embolized leaf veins to the total number of visible leaf veins in the optical sensors and expressed as a percentage. This approach was adopted because it standardizes all embolism events between values of 0 and 100, regardless of leaf size or the number of visible vascular bundles, However, we caution that embolism curves measured using this method are unlikely to represent proportional declines in hydraulic conductance (Cardoso, Batz, and McAdam 2020; Venturas et al. 2019).

### 2.4 Gas Exchange and Chlorophyll Fluorescence

Two LI‐6400/XT Portable Photosynthesis Systems (LI‐COR Biosciences, Lincoln, Nebraska, USA) with 6400‐40 fluorometer chambers were attached to Leaves 13 and 8. After Leaf 8 senesced, the sensor head attached to this leaf was moved up to Leaf 10 (Figure 1). Measurements of gas exchange as well as maximal (*F*_m_′) and minimal (*F*_o_′) yields of chlorophyll fluorescence were taken at 15‐min intervals from the same leaf position using the ‘Auto Log 2′ function with the chamber set to replicate the prevailing light intensity (192 ± 34 µmol photons m^−2^ s^−1^ for Leaf 8; 265 ± 13 µmol photons m^−2^ s^−1^ for Leaf 10; 432 ± 35 µmol photons m^−2^ s^−1^ for Leaf 13). Photosynthetic efficiency was calculated as (*F*_m_′ − *F*_o_′)/*F*_m_′ (Schreiber, Schliwa, and Bilger 1986). Plant transpiration was calculated from changes in whole‐plant mass, which were measured using digital balances (Adam CBK 70 A, Adam Equipment Inc., Oxford, CT, USA) and logged every 30 s. To minimize changes in mass due to substrate evaporation, the tops of pots were wrapped with white plastic (visible in Supporting Information S1: Figure S1). Transpiration measurements were obtained from the instrumented plant and up to six accompanying plants.

### 2.5 Statistical Analysis

Leaf water potential was estimated from individual leaf water potential measurements taken from companion plants and predicted using the time after stomatal closure with linear least squares regression with the ‘lm’ function in R (R Core Team 2021; Supporting Information S1: Figure S2). Increasing embolism and decreasing gas exchange and chlorophyll fluorescence parameters with declining water potential were fit with local polynomial regression models using the ‘loess’ function in R. In each case, 12%, 50% and 88% declines in leaf function were determined, and standard deviations were calculated across the four cohorts using cohort mean values (Table 1). Relationships between embolism accumulation and recovery of photosynthesis after re‐watering were evaluated with ordinary least squares regression using the ‘lm’ function in R. Normalized values of leaf embolism, stomatal conductance, net CO_2_ assimilation and photosynthetic efficiency were fit with 2‐parameter Weibull functions from the ‘drc’ R package (Ritz et al. 2015).

**Table 1:** Water potentials (MPa) corresponding to 12% (P_12_), 50% (P_50_) and 88% (P_88_) of xylem embolism in Leaves 8–13, loss of CO_2_ assimilation, loss of stomatal conductance and loss of photosynthetic efficiency in Leaves 8 and 13.

	P_12_	P_50_	P_88_
Embolism (Leaf 13)	−1.76^a^	−2.28^a^	−2.37^a^
Embolism (Leaf 12)	−1.79 (−1.60, −1.98)^b^	−2.16 (−1.91, −2.41)^b^	−2.33 (−2.18, −2.48)^b^
Embolism (Leaf 11)	−1.85^a^	−2.48^a^	−2.74^a^
Embolism (Leaf 10)	−1.70 (−1.55, −1.84)^b^	−2.13 (−2.00, −2.26)^b^	−2.56 (−2.52, −2.61)^b^
Embolism (Leaf 9)	−1.42 ± 0.09	−1.65 ± 0.20	−1.87 ± 0.20
Embolism (Leaf 8)	−1.25 ± 0.22	−1.34 ± 0.22	−1.52 ± 0.15
CO_2_ assimilation (Leaf 13)	−1.13 ± 0.04	−1.23 ± 0.03	−1.44 ± 0.06
CO_2_ assimilation (Leaf 8)	−1.12 ± 0.08	−1.2 ± 0.09	−1.31 ± 0.09
Stomatal conductance (Leaf 13)	−1.13 ± 0.04	−1.23 ± 0.03	−1.44 ± 0.06
Stomatal conductance (Leaf 8)	−1.12 ± 0.08	−1.2 ± 0.09	−1.31 ± 0.09
Photosynthetic efficiency (Leaf 13)	−1.14 ± 0.03	−1.29 ± 0.03	−2.07 ± 0.19
Photosynthetic efficiency (Leaf 8)	−1.16 ± 0.08	−1.26 ± 0.1	−1.41 ± 0.08

*n* = 4 mean and standard deviation unless indicated.

^a^ *n* = 1 calculated value.

^b^ *n* = 2 mean and range.

## 3 Results

### 3.1 Onset of Leaf Xylem Embolism During Dry Down

**Figure 2:**
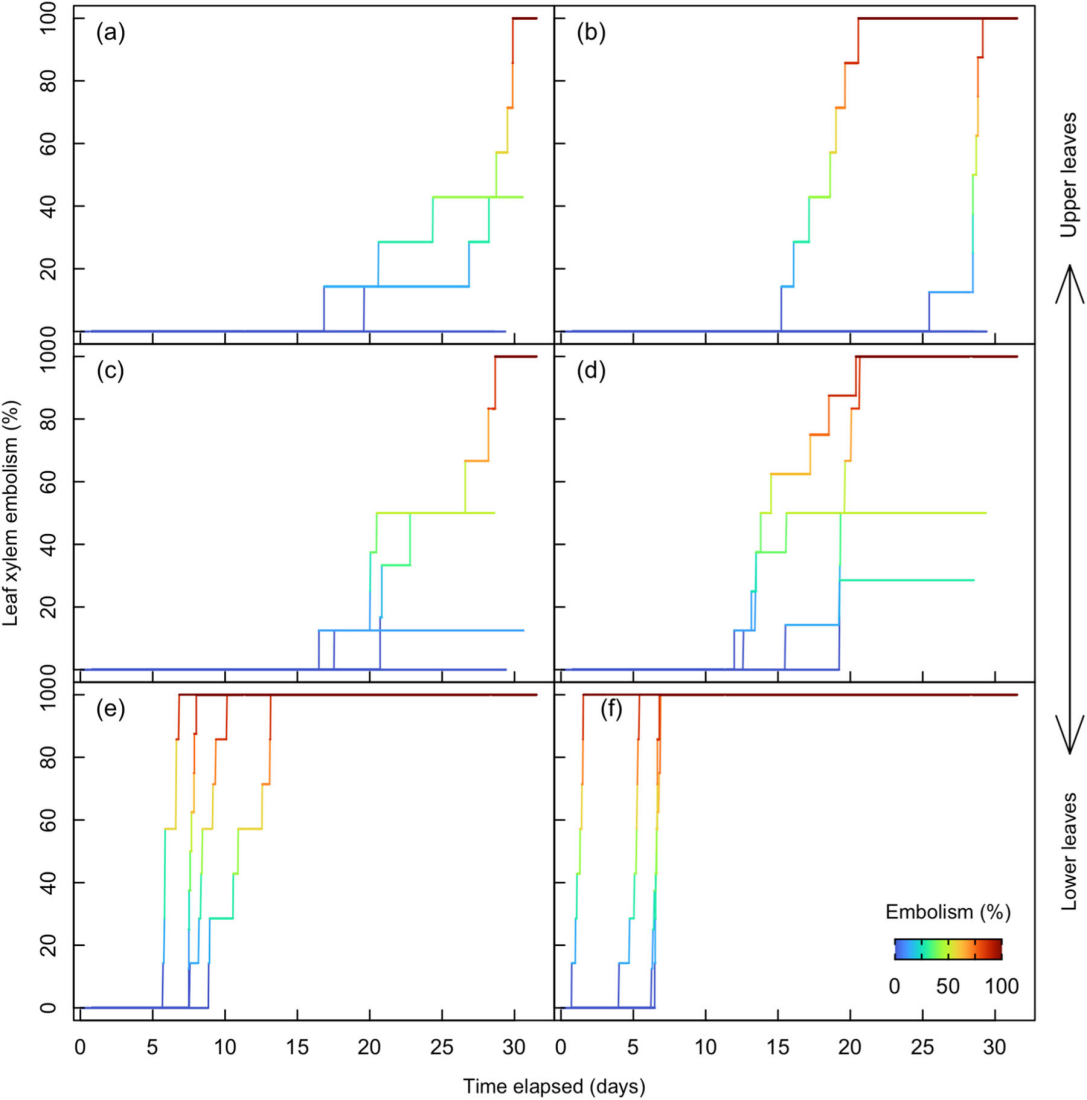
The relationship between xylem embolism in the 13th (a), 12th (b), 11th (c), 10th (d), 9th (e) and 8th (f) leaves and time elapsed. Symbols are coloured by the percentage of leaf vein bundles with embolism within each leaf. *n* = 4 plants. [Color figure can be viewed at wileyonlinelibrary.com]

**Figure 3:**
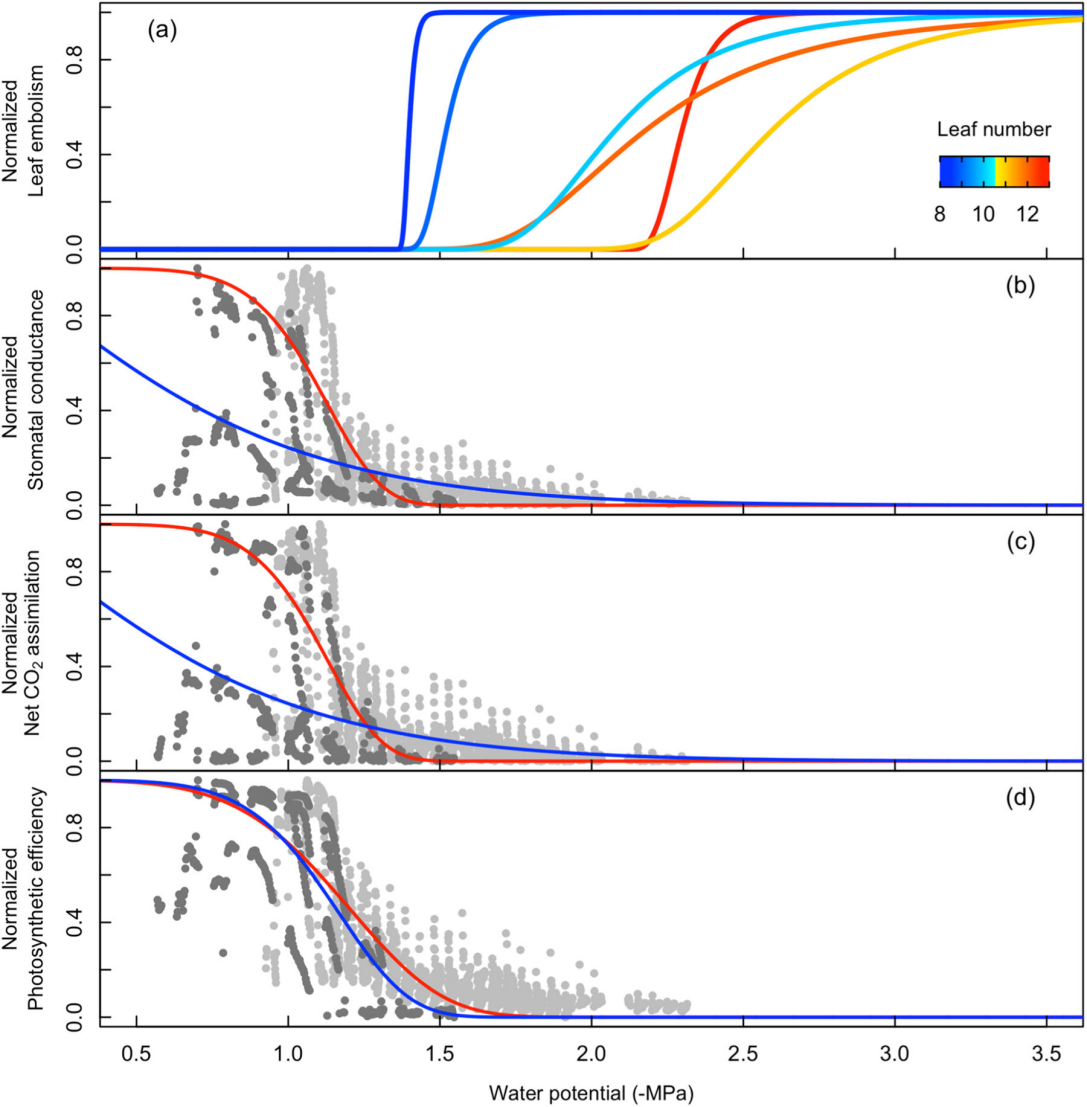
Fitted 2‐parameter Weibull functions of leaf embolism with respect to water potential for the six fully expanded leaves (a) coloured by leaf number. Normalized values of stomatal conductance (b), normalized net CO_2_ assimilation (c) and normalized values of photosystem II efficiency (d) plotted against water potential. Data are fit with 2‐parameter Weibull functions, fitted to Leaf 8 (blue) and leaf 13 (red). Dark grey symbols represent normalized values from Leaf 8 and light grey symbols represent normalized values from Leaf 13. Replication is as shown in Table 1; rows 1–6 (a), *n* = 4 (b–d).

Initial embolization events in the upper leaves of maize started ca. Day 15 (Figure 2) when water potential was ca. −1.7 MPa (Table 1, Figure 3a and Supporting Information S1: Figure S3). In cases where water was withheld longer than 20 days, all upper leaves had some embolism before Day 30 (Figure 2a–c). After Day 10 of the dry down, Leaf 8 had begun to embolize and progressed to 100% before Day 20 (Figure 2f). Leaf 9 was fully embolized within 15 days (Figure 2e). In contrast, the upper leaves (Leaf > 11) did not continue to embolize after plants were re‐watered (Figure 2d). Leaves 11 through 13 behaved similarly after the onset of embolism, exhibiting a gradual accumulation of embolism, resulting in a wider range of water potentials associated with increasing embolism (Figure 3a), in contrast to Leaves 8 and 9, which desiccated much more rapidly and were fully embolized earlier in the dry down. Given these differences in leaf senescence, there was notable variability in the timing and degree of embolism across leaves and replicates. Little embolism was observed in the upper leaves, while Leaf 8 and Leaf 9 were found to embolize (Figure 2e,f) and senesce within the first 10 days of the dry down. The first three cohorts were re‐watered when embolism was observed in some veins of the upper leaves (approximately 20 days into the dry down), whereas the final cohort was dried until embolism was observed in all leaf veins in all upper leaves. This contrast between upper and lower leaves is further reflected by the leaf water potential corresponding to initial embolization events (P_12_), predicted to be Ψ_leaf_ < −1.7 MPa for Leaf 13, whereas in lower leaves, 8 and 9, P_12_ was predicted to be Ψ_leaf_ > −1.5 MPa (Table 1 and Figure 3a).

### 3.2 Decline of Transpiration and Photosynthesis During Dry Down

**Figure 4:**
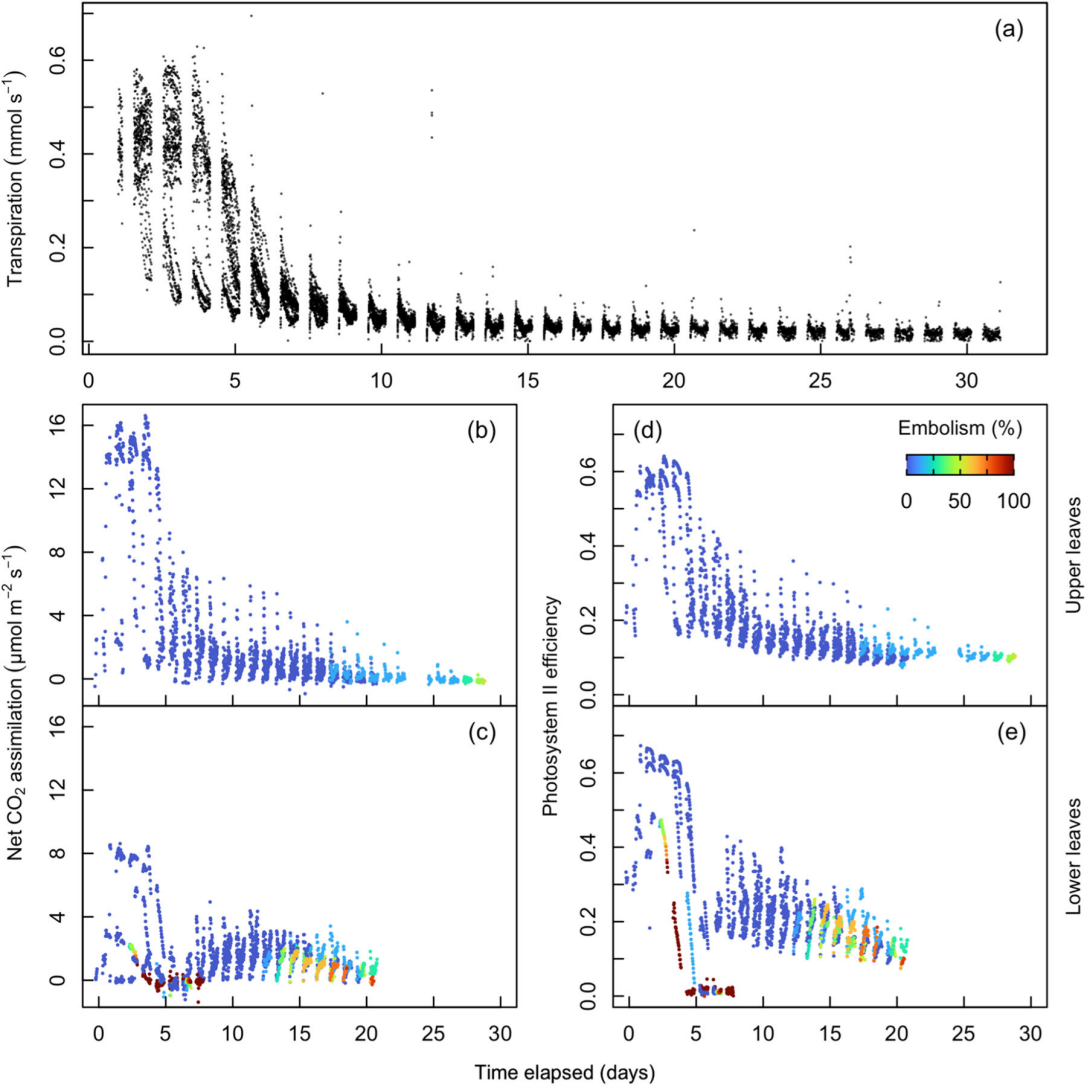
Whole‐plant transpiration rate (a) and CO_2_ assimilation rate (b, c) and photosynthetic efficiency (*F*_v_′/*F*_m_′) (d, e) in upper (b, d) and lower (c, e) collared maize leaves during the dry down period. For (b–e), symbols are coloured by the percent of major vein bundles with embolism within each leaf (see Figure 2). *n* = 24 plants for (a) and *n* = 4 plants for (b–e). [Color figure can be viewed at wileyonlinelibrary.com]

Transpiration was sustained at maximal levels before declining at approximately Day 5 and incrementally decreasing each subsequent day (Figure 4a). Net CO_2_ assimilation in the upper leaves (Leaf 13) declined from maximum values within 5 days of dry down (Figure 4b) when water potential was ca. −1.1 MPa (Table 1, Figure 3c and Supporting Information S1: Figure S3). Matched by declines in daytime photosystem II (PSII) efficiency and eventually reaching near‐zero levels after Day 20 (Figure 4d), at which point water potential was ca. −2 MPa (Table 1 and Figure 3d). In the lower leaves (Leaves 8 and 10), net CO_2_ assimilation, stomatal conductance and photosystem II efficiency declined in concert to a minimal level before Day 5 (Figure 4c) at a water potential of ca. −1.3 MPa (Table 1 and Figure 3b–d). The senescence of Leaf 8 was noted by the sharp decline in PSII efficiency to 0 at roughly 5 days into the dry down period (Figure 4e). At this point, the LI‐6400/XT gas exchange system was moved from Leaf 8 up to Leaf 10, where assimilation and PSII efficiency had reached a steady, but depressed, state for the duration of the dry down until the desiccation of the leaf tissue, at roughly 20 days into the dry down (Figure 4d,e). Leaf functional traits were found to be substantially diminished before the onset of embolism in every leaf measured (Figure 3).

### 3.3 Recovery of Transpiration and Photosynthesis

**Figure 5:**
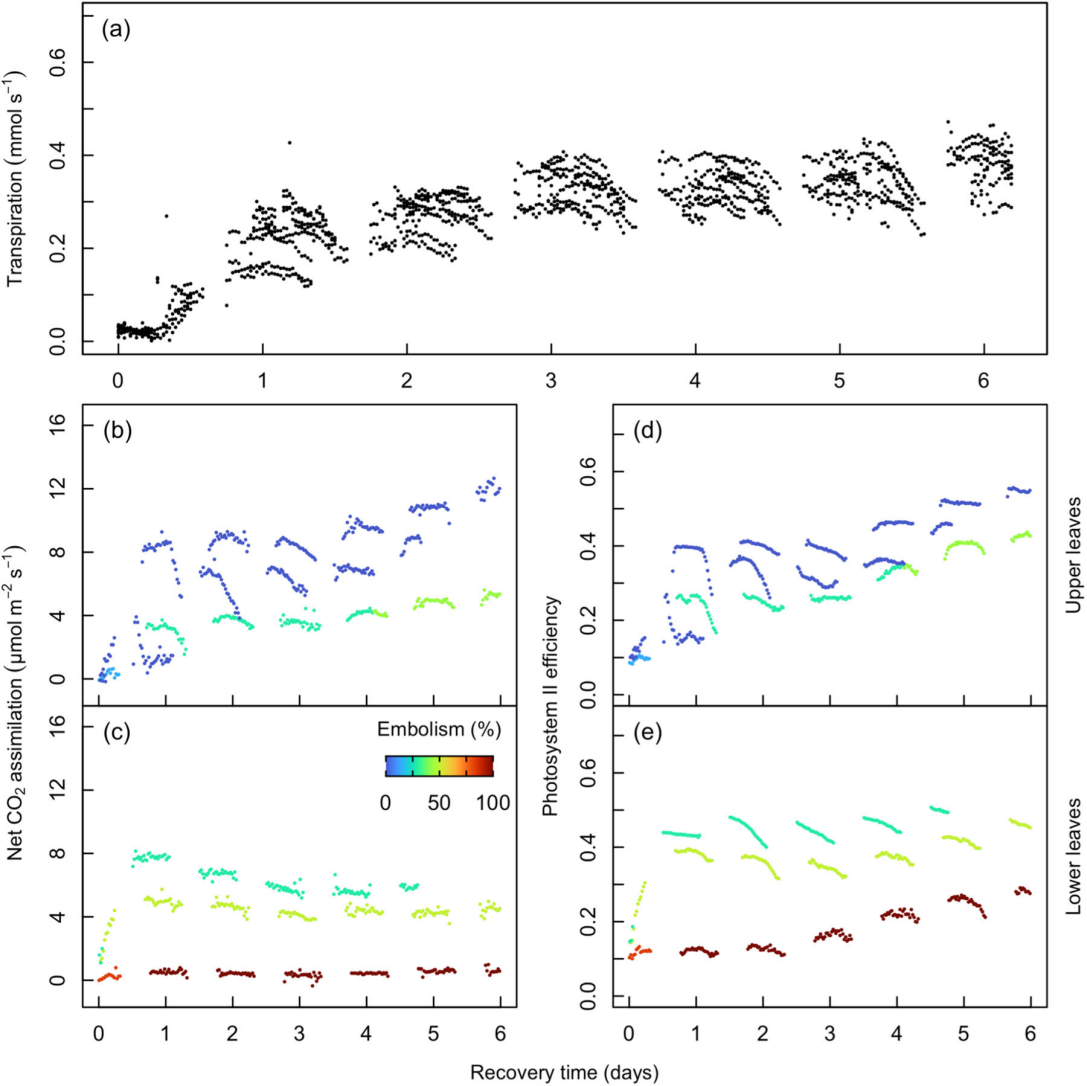
Measurements of whole‐plant transpiration rate (a), net CO_2_ assimilation rate in the upper (Leaf 13; b) and the lower leaf (Leaf 10; c), and photosystem II efficiency (*F*_v_′/*F*_m_′) in upper (Leaf 13; d) and lower (Leaf 10; e) leaves for 6 days after re‐watering. Symbols are coloured by the percent of major veins with observed embolism within each leaf (see Figure 2). *n* = 12 plants for (a) and *n* = 3 plants for (b–e). [Color figure can be viewed at wileyonlinelibrary.com]

Transpiration was observed to recover to a steady state the day after water was added to pots, recovering between 52% and 107% (mean = 79.0%) of average pre‐stress values (Figure 5a). Net CO_2_ assimilation and PSII efficiency were measured for Leaves 10 and 13. The upper leaf accrued up to a 43% reduction in the number of functional leaf veins during the dry down and recovered to a functional steady state 2 days after watering resumed despite the existence of embolism. During recovery, CO_2_ assimilation to 20% and 57% (mean = 43%) of pre‐stress values was aligned with embolism severity (*R* ^2^ = 0.93; *p* < 0.001; Figure 5b). PSII efficiency recovered between 59% and 85% (mean = 75.6%) of pre‐stress values, and similar to CO_2_ assimilation, was also correlated with embolism severity (*R* ^2^ = 0.88; *p* < 0.001; Figure 5d). Nighttime measurements of PSII efficiency for the upper leaf measured initially were 0.72 ± 0.04, declined to 0.46 ± 0.05 before watering plants and recovered to 0.64 ± 0.03 averaged over the last 3 days of the recovery period (Supporting Information S1: Figure S4). However, we note that because one of two gas exchange sensor heads was moved to a higher leaf after Leaf 8 senesced, lower leaf measurements were also taken under slightly higher light intensity (192 µmol photons m^−2^ s^−1^ on Leaf 8 vs. 218 µmol photons m^−2^ s^−1^ on Leaf 10).

Of the plants that were re‐watered, embolism was observed in 29% to 100% of vascular bundles in Leaf 10, the lowest leaf that did not desiccate completely during the dry down. CO_2_ assimilation and PSII efficiency returned to a steady state 1 day after plants were re‐watered (Figure 5c,e). CO_2_ assimilation and photosynthetic efficiency (Figure 5c,d) recovery trajectories were separated by the degree of embolism accumulated in the leaf, with differences being greater in the lower leaves (Figure 5c,e). Completely embolized leaves had little to no recovery of CO_2_ assimilation and severely reduced recovery of PSII efficiency (Figure 5c,e).

### 3.4 Inter‐Vein Distance

**Figure 6:**
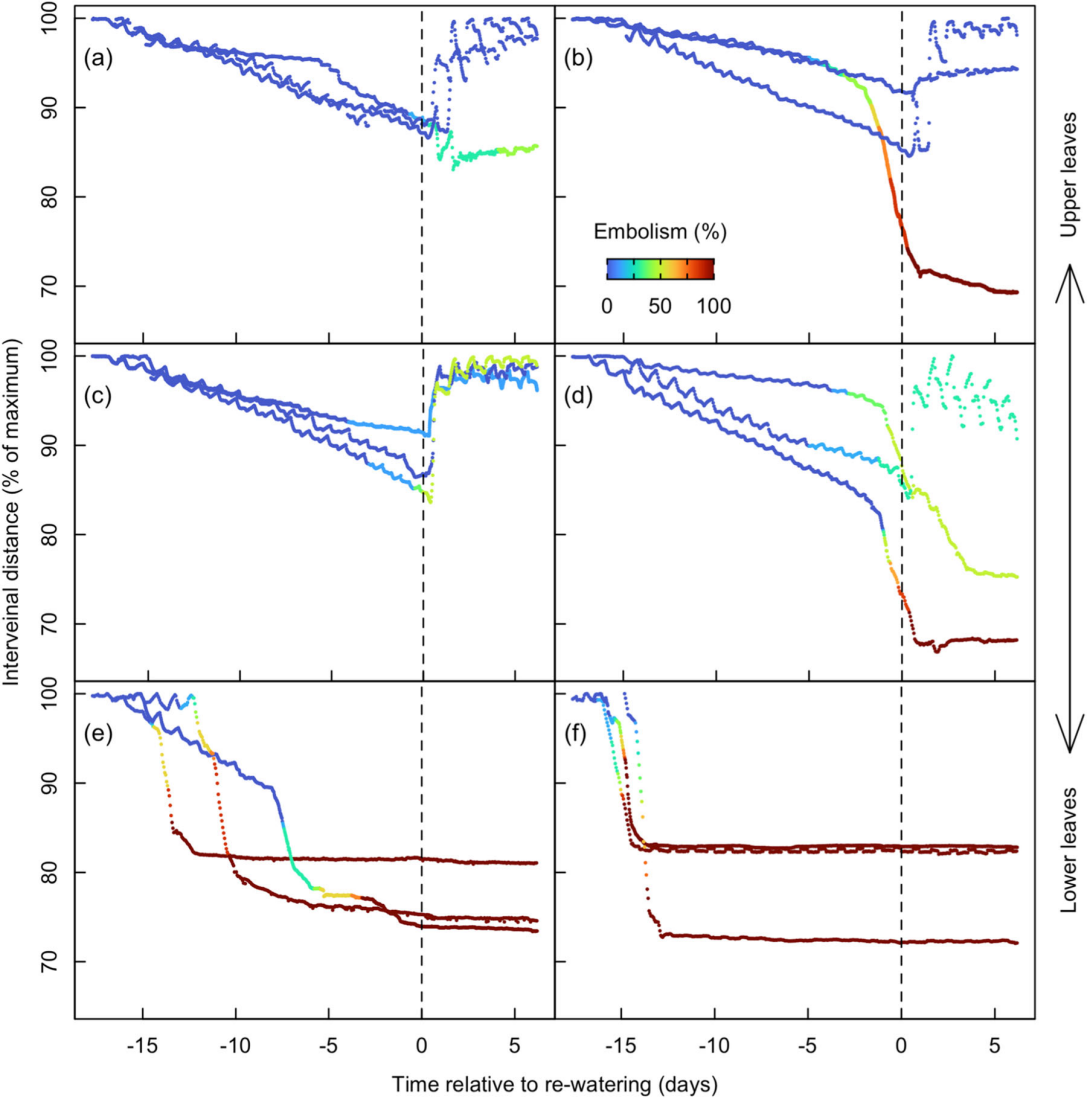
Inter‐vein distance in the 13th (a), 12th (b), 11th (c), 10th (d), 9th (e) and 8th (f) collared leaves from the base. Vertical dashed lines indicate the time at which plants were re‐watered (i.e., the end of the dry down period and the start of well‐watered period). Symbols are coloured by the percentage of leaf vein bundles with embolism within each leaf (see Figure 2). *n* = 3 plants. [Color figure can be viewed at wileyonlinelibrary.com]

In all leaves, the distance between veins on either side of the midrib decreased when water was withheld, and this was represented as a normalized fraction of the initial distance (Figure 6). Upper leaves experienced embolism much later into the dry down and were able to recover near initial values (Figure 6a). Most leaves that had experienced some embolism did respond to re‐watering and recovered to near‐maximal values within two nights of well‐watered conditions (Figure 6b–d). In leaves where all visible veins were observed, the inter‐vein distance reached a minimal value and did not respond to re‐watering (Figure 6d–f). Leaves 8 and 9 reached minimal values shortly after the complete embolization of the leaf's veins (Figure 6e,f). In most cases, the inter‐vein distance would decline by roughly 15%–30% of initial values before localized leaf desiccation. In cases where minimal embolism was observed, the decline in inter‐vein distance was less than 20%, these leaves were able to recover to near initial inter‐vein distance upon re‐watering (Figure 6a–d).

## 4 Discussion

This study evaluated embolism formation non‐destructively during a dry down of intact maize plants and assessed the physiological consequences of accumulated leaf embolism across the canopy during recovery. We categorized several physiological leaf traits spatially and temporally in response to declining leaf water potential after withholding water and then subsequent recovery after re‐watering. The sequence of decline in physiological traits during dry down was aligned with our expectations, becoming substantially depressed at water potentials below ca. −1.4 MPa. Notably, there was a very long time period (ca. 15 days) between stomatal closure and embolism onset in the upper (but not lower) leaves, despite stomatal conductance and net CO_2_ assimilation becoming meaningfully reduced at similar water potentials for both leaf positions. During the dry down period, Inter‐vein distance steadily decreased in all leaves until reaching a critical point around the time of moderate embolism (ca. 30%). After which, the decrease in inter‐vein distance accelerated until stabilizing again when leaf tissue had become completely desiccated. In both the upper leaves and lower leaves, the amount of accumulated embolism influenced the recovery of transpiration, photosynthesis and PSII efficiency, highlighting the alignment between hydraulic system integrity and leaf physiological function following a prolonged experimental drought.

### 4.1 Spatial and Temporal Patterns of Stomatal Closure and Xylem Embolism

In both the upper and lower leaves, initial declines in leaf‐level physiological processes occurred at a similar time and water potential during the dry down, corresponding to the depletion of available soil water (Brodribb and Holbrook 2003; Gleason et al. 2017b). However, there was a pronounced time lag and slight pressure difference between the upper and lower leaves when large declines in photosynthesis and stomatal conductance were observed (i.e., P_88_). The range of leaf water potentials (−1.3 to −2.5 MPa) representing a 50% decline in the number of functional leaf veins (P_50_) falls within the range reported from studies using other methods, that is, expressing hydraulic decline as the loss of hydraulic conductance/conductivity (Cochard 2002; Gleason et al. 2017b; Li, Sperry, and Shao 2009). Optical vulnerability assessments do not always accurately reflect the extent of leaf xylem embolism because leaf minor and transverse veins are often obstructed by bundle sheath chloroplasts. This limited our analysis to leaf major veins which allowed us to assess the relative timing of embolism formation but also limited our capacity to address the full spectrum of leaf hydraulic impairment. While embolism assessments using the optical method should not be interpreted as proportional losses of hydraulic conductance, they align reasonably well with reported values. For instance, stem‐specific hydraulic conductivity declined by over 60% at leaf water potentials below ca. −2 MPa, and partially recovered after plants were watered (Gleason et al. 2017b). Excised leaf midribs lost 50% of xylem conductance at −2.5 MPa (Cochard 2002). Most hydraulic methods disregard the time component when creating vulnerability curves, often using excised leaf material to accelerate drying time. Similarly, a recent evaluation of the centrifuge method reports that shorter spin times are associated with underestimation of embolism (Silva et al. 2024). We propose that this disregard for time when estimating embolism vulnerability also disconnects vulnerability assessments from potential acclimation processes (i.e., osmotic adjustment and water storage tissue dynamics) that would normally occur during natural drought events and is a possible explanation for the wide range of vulnerability found between lower and upper canopy leaves in our experiment. The large variability in leaf embolism that we observed may also have resulted from hydraulic isolation of individual leaves. Measured plants had exhausted available soil water before Day 10 (ca. −1.5 MPa; Gleason et al. 2017a). It is at this point the diurnal fluctuations in inter‐vein distance become minimized along with other leaf functional traits, and when leaf desiccation began to follow a bottom‐up pattern, that is, leaves closer to the vegetative whorl embolized later and at lower water potentials, similar to a previous report on maize (Sun et al. 2015). The preservation of young upper leaves may benefit species during acute drought periods by reducing the maintenance and transpirational costs of less‐productive older leaves, and thereby lengthening the time to critical hydraulic failure in the upper canopy (Acevedo et al. 1979; Aparicio‐Tejo and Boyer 1983; Blackman et al. 2016; Gleason et al. 2014). Considering that the youngest and most productive leaves at the top of the canopy (Leaves 10–13) are also most proximal to the developing infructescence, it would be advantageous for these leaves to remain functional for grain filling purposes (Westgate and Boyer 1985; Xu, Zhou, and He 2021). The idea of selection favoring upper canopy leaves (see also Hochberg et al. 2017) is also aligned with the segmentation hypothesis first proposed by Zimmermann (1983) and necessitates further investigation of the hydraulic isolative properties of grass leaves and other monocotyledonous species.

### 4.2 Recovery of Leaf‐Level Transpiration and Photosynthesis Corresponds to the Extent of Embolism

The recovery of leaf physiological traits was influenced by the degree of embolism accumulation, reflecting variation in the sustained loss of hydraulic conductance. We did not observe a reversal of leaf embolism upon re‐watering, which is consistent with previous findings (Cardoso, Batz, and McAdam 2020; Johnson, Jordan, and Brodribb 2018; Tonet, Brodribb, and Bourbia 2024). After re‐watering, the function of recovered leaves was hindered, where average CO_2_ assimilation and stomatal conductance were only ca. 60% of their initial, pre‐stressed, values. This lasting depression of leaf‐level traits could possibly be the result of compounding reductions in hydraulic conductivity in tissues upstream of leaves (e.g., root death, loss of capacitance tissues, stem embolism), although this hypothesis was not tested.

Plants were able to resume growth in the form of expanding internodes and resumed emergence of developing leaves which were confined to the vegetative whorl before re‐watering. Regardless of the minimum leaf water potential plants experienced during the dry down, growth points within the stem were seemingly conserved during the dry down, avoiding desiccation (Bramley et al. 2015; Johnson, Jordan, and Brodribb 2018). We did not evaluate stem‐specific embolism or conductivity declines so we cannot assess whether maize stems were actually less vulnerable than leaves and we remain uncertain if there may have been stem embolism at all. However, partial recovery of stem‐specific conductivity and internode embolism has been observed in excised maize stems when water potentials returned above −0.3 MPa, this may be true for maize stems where inner compartments are better isolated from atmospheric conditions. (Gleason et al. 2017b). In our case, it is possible that leaf water potential did not recover to the level necessary for refilling to occur within the recovery period, or that our prevailing conditions were unfavourable for leaf conduit refilling (i.e., high relative humidities, low irradiance and low VPD would be more conducive). Additionally, prolonged exposure to near embolism inducing pressures may have caused tissue degradation, consistent with observed trends linking desiccation to sustained photosynthetic efficiency declines (Trueba et al. 2019).

The spatial variation of plant leaves/compartments remaining relatively well hydrated is also reflected in the contrasting leaf water potentials between lower versus upper leaves (Supporting Information S1: Figure S2; Sun et al. 2015; Tang and Boyer 2008). This compartmentalized water loss likely aids plant resilience by placing meristematic and desiccation sensitive reproductive structures at a higher priority than leaves (Bassetti and Westgate 1993). This would permit seed development in the absence of photosynthesis by the movement of stored carbon or by aiding post‐drought recovery and growth from the remobilization leaf compounds sourced from senescent lower leaves (Field 1983; Jurgens, Johnson, and Boyer 1978). In contrast to the plausible importance of the upper canopy in annual grasses like maize, at least among perennial grasses, leaf embolism and water transport capacity may be poor indicators of plant mortality (Ocheltree, Nippert, and Prasad 2016).

### 4.3 Inter‐Vein Distance Dynamics Mimic Leaf Water Status

Here, we found that the rate of shrinkage, estimated here as inter‐vein distance, accelerated rapidly after the onset of xylem embolism. This acceleration suggests that embolism obstruction upstream from the camera (measuring inter‐vein distance) resulted in marked restriction of water delivery to the lamina and, thus, the relative water content of the lamina (Abate et al. 2021; Brodribb et al. 2021; Tonet et al. 2023). However, this decline in inter‐vein distance was only reversible in leaves experiencing less than 50% loss of functional leaf veins (cf. Brodribb et al. 2021; Figure 6). Likewise, the onset of embolism did not always lead to the acceleration of leaf shrinkage, and the time between embolism onset and the acceleration of leaf shrinkage varied widely. In the present study, leaf shrinkage provided a continuous, nondestructive estimate of internal water status from full hydration until complete desiccation and tissue death. Considering that pressure chamber measurements and stem psychrometers become increasingly difficult at low volumetric water contents (e.g., after the onset of embolism), leaf shrinkage may serve as a useful proxy for internal water status of herbaceous, non‐woody tissues when calibrated using a robust series of pressure chamber or psychrometer measurements (Bourbia et al. 2023; Gleason et al. 2024).

## 5 Conclusions

Our findings show that maize leaves can partially recover function in the presence of considerable sustained embolism, and the magnitude of this recovery after re‐watering is aligned with the degree of embolism. Similar to a previous study on wheat, we did not observe embolism refilling in leaves after re‐watering (Johnson, Jordan, and Brodribb 2018). Although conduit refilling may occur when adjacent tissues are well‐hydrated or when prevailing environmental conditions favour refilling (Hwang, Ryu, and Lee 2016; Ryu, Hwang, and Lee 2016), we did not observe refilling in maize leaves. Variation in water loss rates among leaves at different positions highlights the dynamic nature of water regulation within the canopy of maize. Additionally, the prolonged depression of CO_2_ assimilation in a laboratory setting may not directly apply to field conditions due to more complex interactions with soil water and fluctuating environmental conditions. The observed recovery of upper canopy leaves post‐embolism emphasizes the importance of hydraulic resistance and compartmentalization in protecting sensitive tissues during desiccation, particularly in grasses that possess a well‐defined culm. Future experiments should focus on examining the long‐term effects of sustained leaf embolism on hydraulic conductivity. This combined approach is necessary to determine whether specific vein orders are more susceptible to embolism and to assess if extraxylary resistances significantly impair the leaf's capacity for water transport. The integration of other methods may lead to the identification of leaf traits (either anatomical or biochemical) that would confer greater embolism resistance for future agricultural cultivars. Prolonged dry down experiments of intact plants offer a more nuanced look into whole plant water dynamics and responses to severe water limitation. These insights enhance our understanding of plant–water relations and highlight the need to consider leaf shrinkage and whole‐plant dynamics in drought stress research, breeding programmes and water management practices.

## Conflicts of Interest

The authors declare no conflicts of interest.

## Supporting information

Supporting information.

Supporting information.

## Data Availability

The data supporting the findings of this study can be found in the supplementary materials of the article. For inquiries or requests for additional data, please reach out to the corresponding author or S.M.G.
