# Supplementary material for: Long‐Term in vivo Observation of Maize Leaf Xylem Embolism, Transpiration and Photosynthesis During Drought and Recovery
Source: Plant Cell Environ. 2025 Feb 3;48(6):4114–25. doi: 10.1111/pce.15414 (PMC12050386; doi:10.1111/pce.15414)
Supplement: Supplementary file 1 — Supporting information. [file PCE-48-4114-s002.docx]

**Supplemental Figure 1**
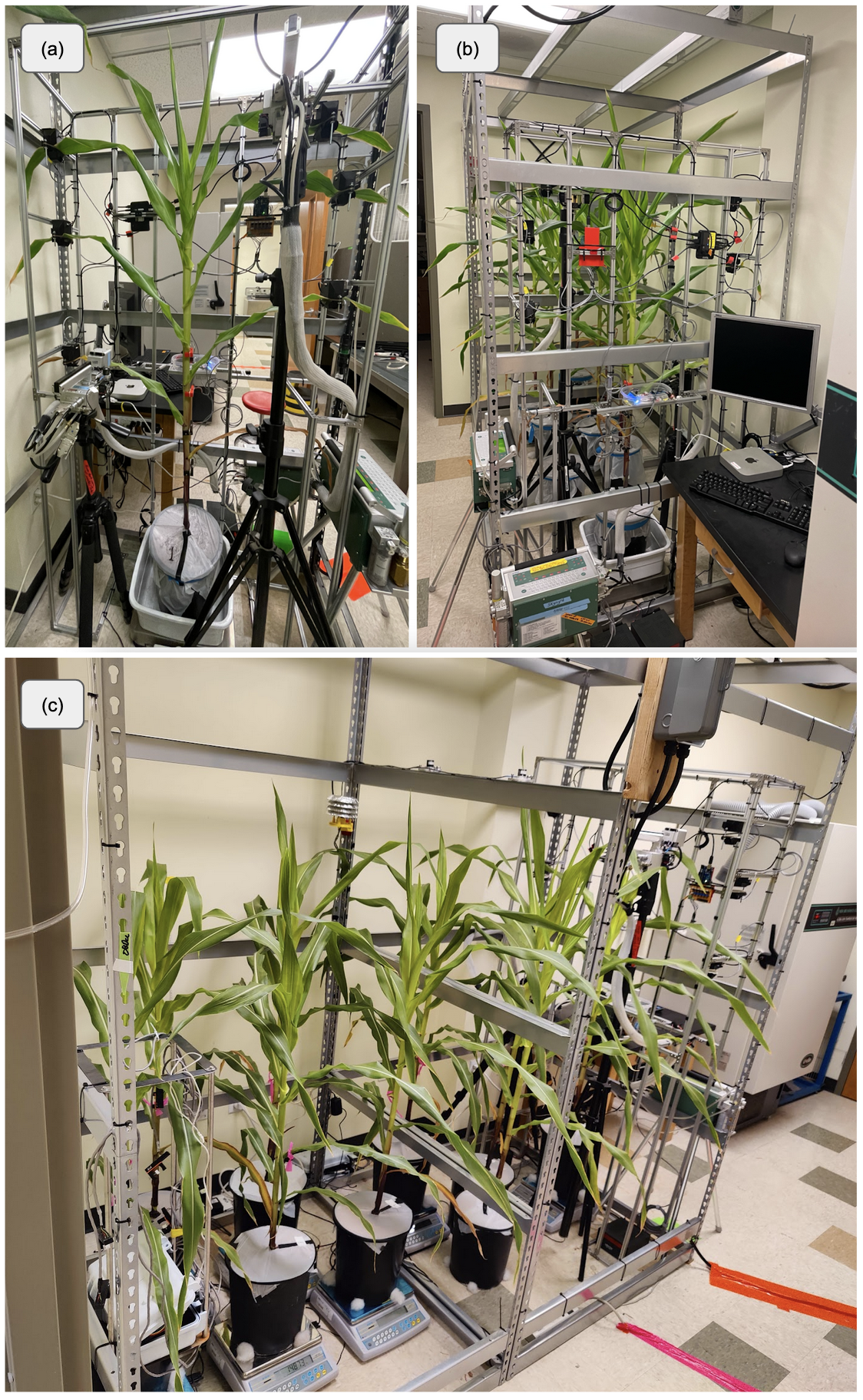


**Figure S1.** Images of the experimental setup. The instrumented plant with accompanying optical sensors placed on leaves 8-13, LI-6400/XT placed on leaves 8 and 13, and balance (a). Lighting placed above plants in the cage and the logging computer (b). Companion plants placed on balances and climatic sensors (c).

**Supplemental Figure 2**

**
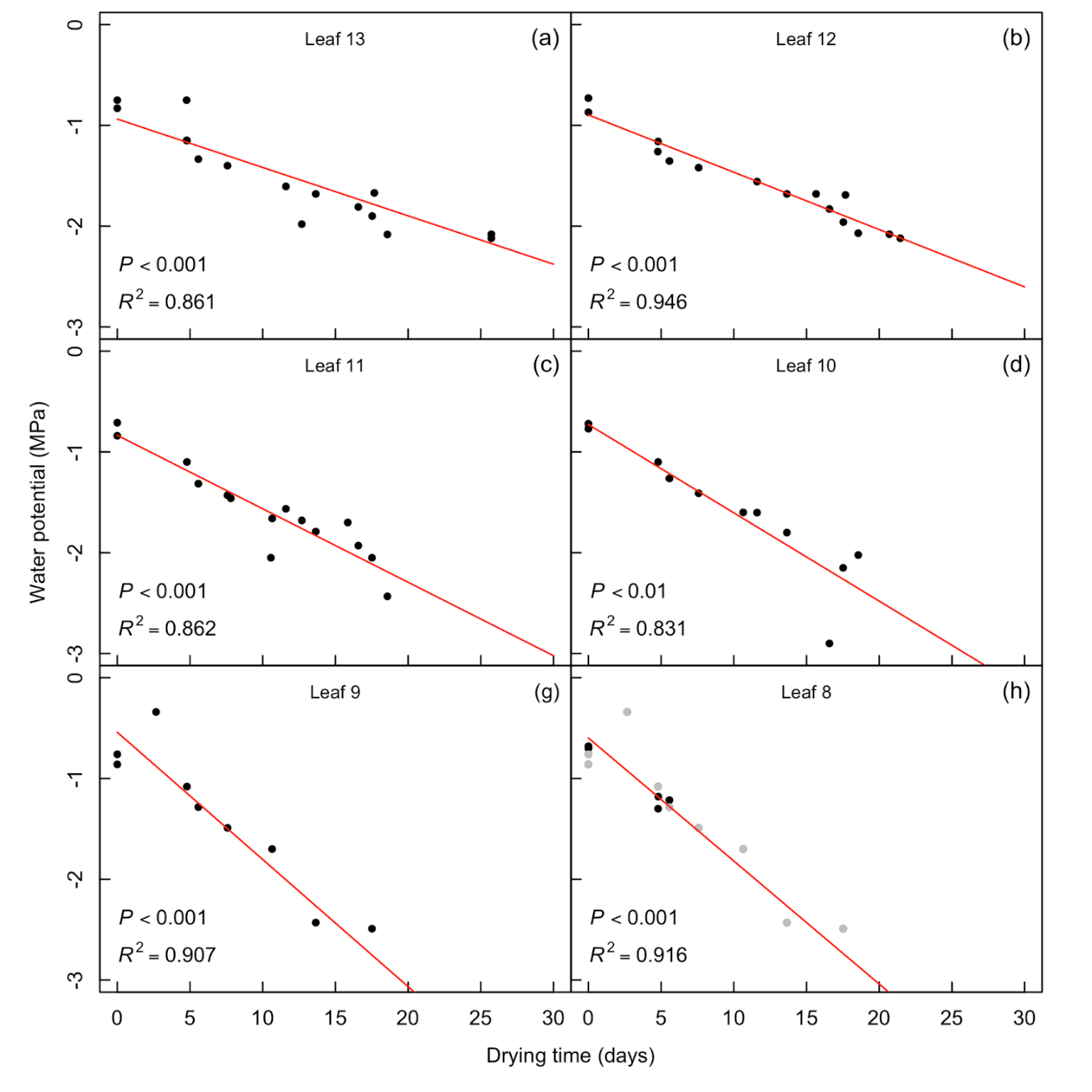
**

**Figure S2.** Measurements of leaf water potential (MPa) taken from companion plants during the dry down period. Measurements are from collared leaves numbered from the base. The 13th (a), 12th (b), 11th (c), 10th (d), 9th (e), and 8th, in black, pooled with leaf 9 measurements, in grey, for better predictions of water potential for this leaf (f). Fitted lines represent predicted water potentials used for individual leaves presented in Table 1. *n = 19* (a), *n = 15* (b), *n = 15* (c), *n = 11* (d), *n = 11* (e), *n = 11* (f) plants.

**Supplemental Figure 3**

**
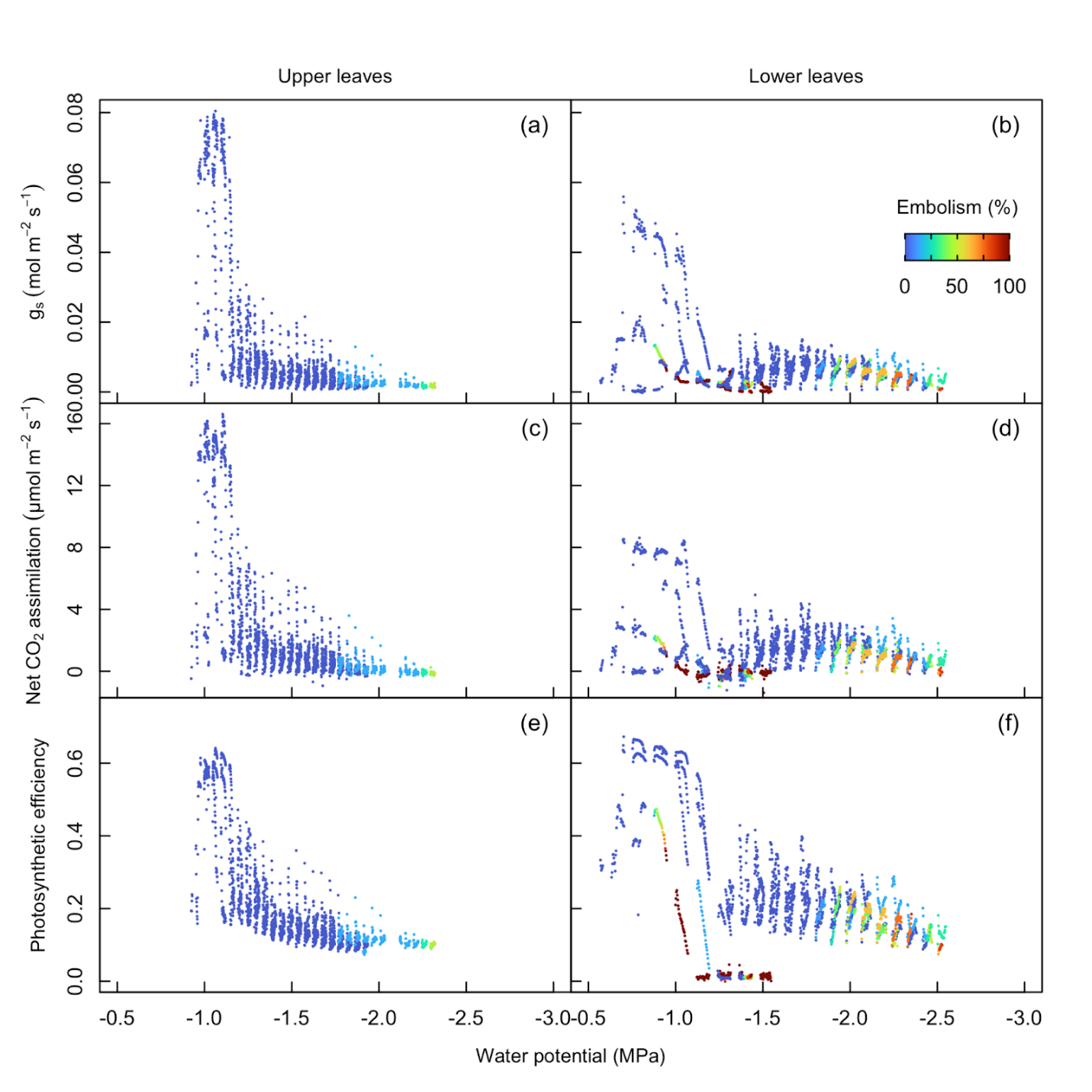
**

﻿**Figure S3.** Measurements of stomatal conductance (a,b), Net CO2 assimilation rate in the upper (leaf 13; c) and the lower leaf (leaf 10; d). Photosystem II efficiency (Fv′/Fm′) in upper (leaf 13; e) and lower (leaf 10; f) plotted with predicted leaf water potential. Symbols are colored by the percent of major veins with observed embolism within each leaf (see Fig. 1) *n = 4* plants.

**Supplemental Figure 4**

**
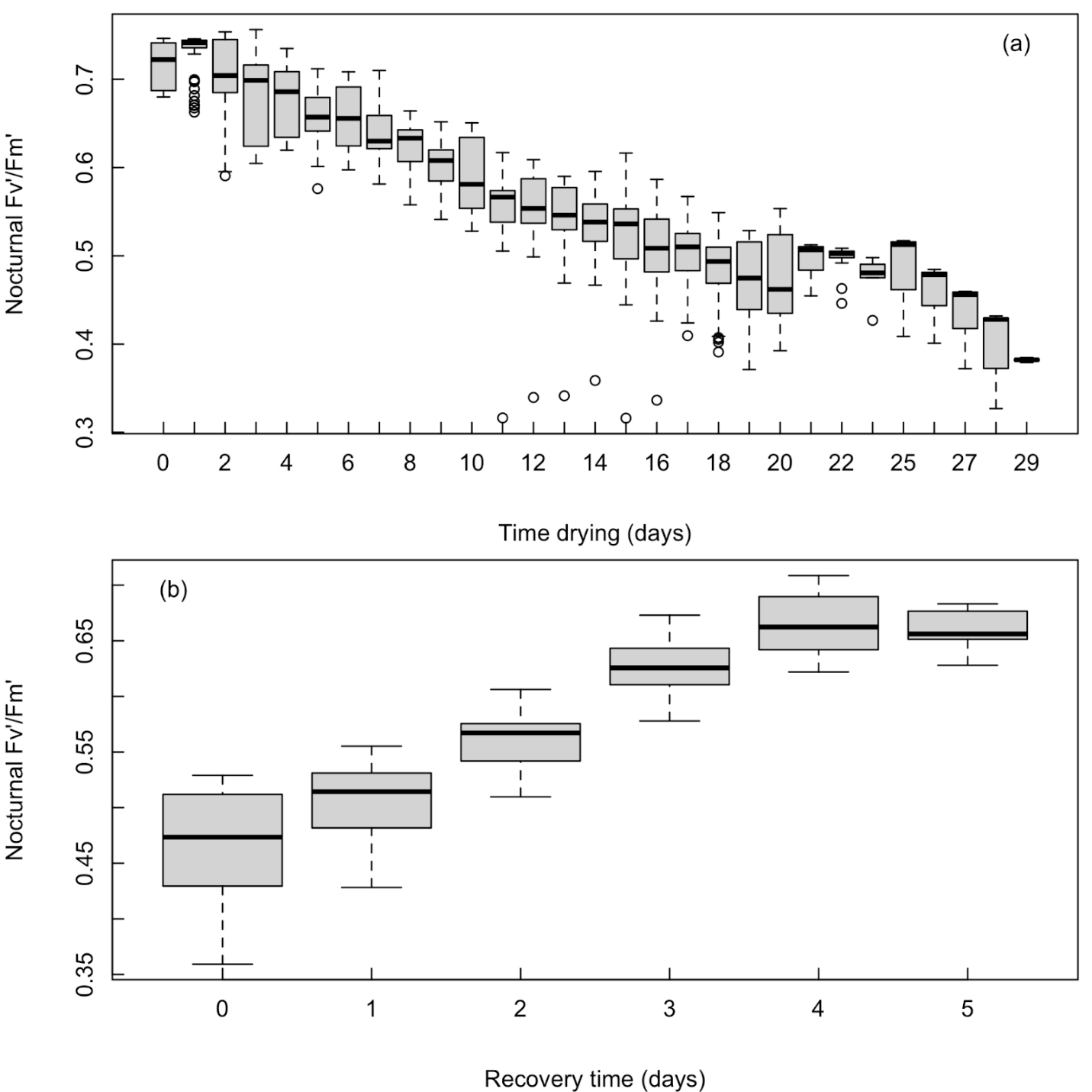
**

**Figure S4.** Nighttime measurements of photosynthetic efficiency measured as Fv’/Fm’ in near darkness (0.64 ± 0.43 µmol photons m^−2^ s^−1^), from leaf 13 for the dry down (a) and recovery (b) period*. n=4* (a) and *n=3* (b) plants.
